# Evaluation of fMRI activation in post-stroke patients with movement disorders after repetitive transcranial magnetic stimulation: a scoping review

**DOI:** 10.3389/fneur.2023.1192545

**Published:** 2023-06-19

**Authors:** Siman Cheng, Rong Xin, Yan Zhao, Pu Wang, Wuwei Feng, Peng Liu

**Affiliations:** ^1^Department of Rehabilitation Medicine, The Seventh Affiliated Hospital, Sun Yat-sen University, Shenzhen, Guangdong, China; ^2^Department of Rehabilitation Medicine, The First Affiliated Hospital, Sun Yat-sen University, Guangzhou, Guangdong, China; ^3^Department of Neurology, Medical University of South Carolina, Charleston, SC, United States

**Keywords:** repetitive transcranial magnetic stimulation, functional magnetic resonance imaging, scoping review, stroke, movement disorders

## Abstract

**Background:**

Movement disorders are one of the most common stroke residual effects, which cause a major stress on their families and society. Repetitive transcranial magnetic stimulation (rTMS) could change neuroplasticity, which has been suggested as an alternative rehabilitative treatment for enhancing stroke recovery. Functional magnetic resonance imaging (fMRI) is a promising tool to explore neural mechanisms underlying rTMS intervention.

**Object:**

Our primary goal is to better understand the neuroplastic mechanisms of rTMS in stroke rehabilitation, this paper provides a scoping review of recent studies, which investigate the alteration of brain activity using fMRI after the application of rTMS over the primary motor area (M1) in movement disorders patients after stroke.

**Method:**

The database PubMed, Embase, Web of Science, WanFang Chinese database, ZhiWang Chinese database from establishment of each database until December 2022 were included. Two researchers reviewed the study, collected the information and the relevant characteristic extracted to a summary table. Two researchers also assessed the quality of literature with the Downs and Black criteria. When the two researchers unable to reach an agreement, a third researcher would have been consulted.

**Results:**

Seven hundred and eleven studies in all were discovered in the databases, and nine were finally enrolled. They were of good quality or fair quality. The literature mainly involved the therapeutic effect and imaging mechanisms of rTMS on improving movement disorders after stroke. In all of them, there was improvement of the motor function post-rTMS treatment. Both high-frequency rTMS (HF-rTMS) and low-frequency rTMS (LF-rTMS) can induce increased functional connectivity, which may not directly correspond to the impact of rTMS on the activation of the stimulated brain areas. Comparing real rTMS with sham group, the neuroplastic effect of real rTMS can lead to better functional connectivity in the brain network in assisting stroke recovery.

**Conclusion:**

rTMS allows the excitation and synchronization of neural activity, promotes the reorganization of brain function, and achieves the motor function recovery. fMRI can observe the influence of rTMS on brain networks and reveal the neuroplasticity mechanism of post-stroke rehabilitation. The scoping review helps us to put forward a series of recommendations that might guide future researchers exploring the effect of motor stroke treatments on brain connectivity.

## Introduction

According to The Global Burden of Diseases, Injuries, and Risk Factors Study (1), stroke accounted for 101 million prevalent cases, 6.55 million deaths, and 143 million disability-adjusted life-years worldwide in 2019. Ischemic and hemorrhagic stroke are the two main types of stroke. Ischemic stroke was defined as an episode of neurological dysfunction resulting from decreased blood flow to a certain area of the brain. In contrast, hemorrhagic stroke was not brought on by trauma but rather by a weak blood vessel that bursts and bleeds into the surrounding brain tissue (2). Stroke is the principal cause of serious disability globally, and movement disorders are one of the most prevalent sequelae of stroke (3). Meanwhile, the recovery of movement disorders after stroke are often incomplete, which cause a major stress on their families and society (4). Stroke results in neuronal death in the directly damaged brain regions. On the other hand, cortical regions remote from directly damaged areas also undergo secondary degeneration or reorganization, leading to widespread changes in the structure and function of the whole brain network (5). In a word, the direct injury effects of motor neurons and their descending axons, as well as abnormal connections remote from the injured lesion, may be important pathophysiological factors for stroke residual effects (6). These changes are closely related to neurological function deficits and subsequent recovery after stroke (7). It is considered that recovery of motor function following stroke depends on neuroplasticity (8). The nervous system's ability to adjust to pressure from the environment, new experiences, and changes—including brain injury—is known as neuroplasticity (9, 10). The development of our knowledge of the neuroplastic changes has inspired researchers to look into methods of anticipating probable post-stroke recovery.

**Figure 1:**
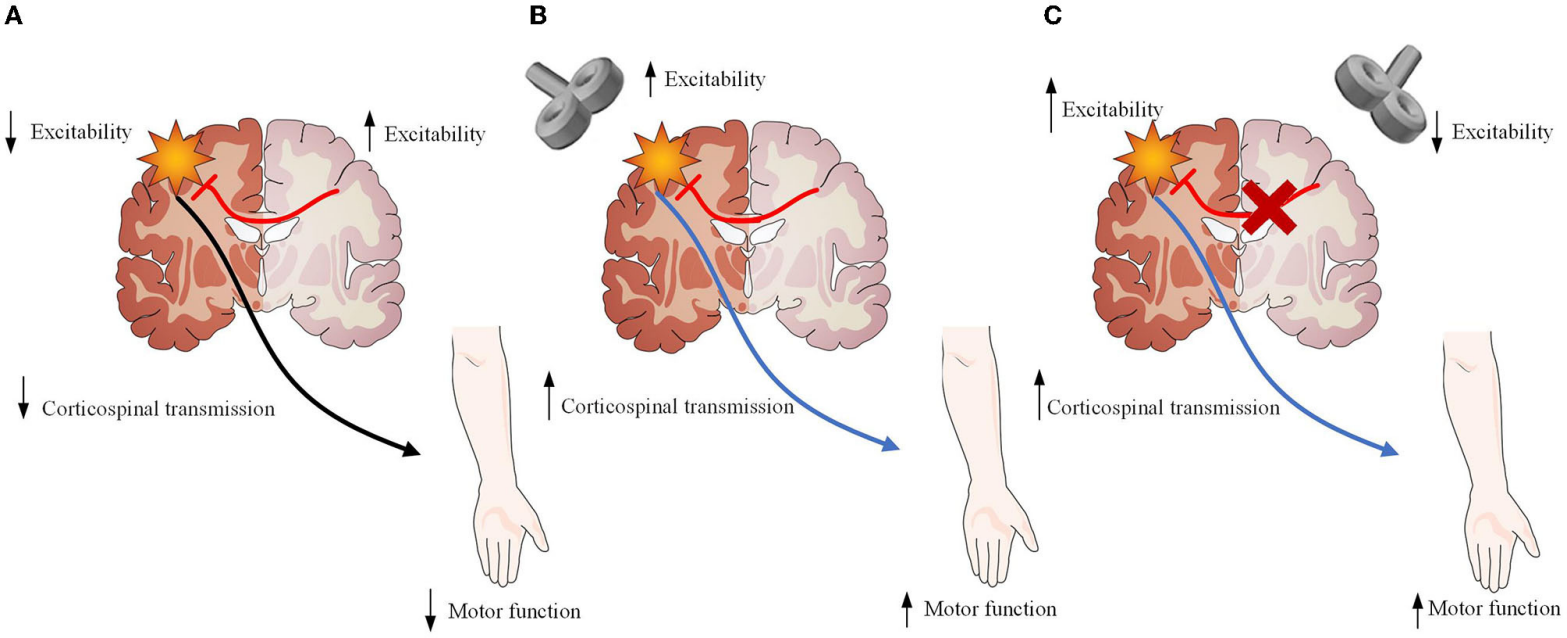
Description of the neurobiological model of rTMS as an treatment for stroke recovery. **(A)** Damage to one hemisphere in stroke results not only from neuronal loss within the affected hemisphere but also from the downregulation of remaining neurons within the affected hemisphere resulting in increased inhibition of the affected hemisphere by the unaffected hemisphere. Both are likely involved in lack of functional recovery. **(B)** High-frequency rTMS applied over the ipsilesional hemisphere strengthens the descending motor pathway, facilitating motor recovery. **(C)** Low-frequency rTMS applied over the contralesional hemisphere reduces inhibitory signals from the contralesional motor cortex, promoting beneficial cortical reorganization and motor recovery. Figure was modified from Servier Medical Art (http://smart.servier.com/), licensed under a Creative Common Attribution 3.0 Generic License. (https://creativecommons.org/Iicenses/by/3.0/) .

Repetitive transcranial magnetic stimulation (rTMS) has already aroused increasingly attention as a tool for modulating stroke-induced abnormal brain network activity and functional connections, which allow the brain to change and adapt to injury following stroke (11). rTMS could improve movement disorders by enhancing the neural plasticity of the brain networks (12). Additionally, its long-lasting neuromodulation beyond the stimulation period could make rTMS suitable for the treatment of movement disorders after stroke (13). High-frequency rTMS (HF-rTMS) on the ipsilesional hemisphere can upregulate the excitatory effects of the ipsilesional cortex, while low-frequency rTMS (LF-rTMS) on the contralesional hemisphere can downregulate excitatory effects of the ipsilesional cortex (14) (Figure 1). They have been widely utilized during the acute, subacute, and chronic phases, and have been proven to restore motor function after stroke (15). Despite the benefits associated with rTMS, such as motor recovery, the mechanisms through which rTMS exerts therapeutic effects remain poorly understood.

Functional magnetic resonance imaging (fMRI) measures changes in blood oxygen levels in the brain, and the blood-oxygen-level-dependent (BOLD) signal evaluates brain activity, with better temporal resolution than PET and SPECT, and superior spatial resolution compared to EEG and MEG (16). People can use fluctuations in the BOLD signal to assess functional connectivity, a method identifying correlation patterns between brain regions (17). fMRI is a non-invasive imaging technique to accurately describe the reorganization of the cerebral cortex, changes in inter-hemispheric balance, and activity changes of the hemispheres (18). fMRI includes task-based fMRI and resting-state fMRI(rs-fMRI). Task-based fMRI is a technique that requires subjects to perform specific tasks during scanning. Researchers have used finger-tapping task-based fMRI to investigate changes in the activation of the sensorimotor network pre- and post-rTMS stimulation (19). rs-fMRI is sensitive to changes in deoxyhemoglobin, which can indirectly reflect changes in neuronal activity (20). Thus, rs-fMRI are applicable to stroke survivors with motor dysfunctions. fMRI has been used to explore the underlying mechanisms of rTMS-mediated neuronal modulation and make us better understand the plasticity in the brain network.

The neural response induced by rTMS is not confined to the stimulated brain area, but can also spread to other cortical regions remote from the stimulated area (21). Hence, rTMS can affect extensive brain functional networks and even whole brain activity (22). Researchers and clinicians have harnessed neuromodulatory effects to promote motor recovery in stroke survivors with the aid of rTMS, possibly owing to alterations in the regions below the stimulation site and where sensory-motor networks are connected (23, 24). Most researches used behavioral and neurophysiological measures to evaluated how rTMS affected stroke motor recovery (25). Nevertheless, they were unable to offer information on brain changes with an outstanding spatial resolution. To date, the neural mechanisms associated with rTMS intervention and their influence on functional connections are rather complicated and poorly understood. From a clinical perspective, a profound understanding of the neural mechanisms underlying recovery is helpful for developing efficient therapy in the future. In order to better understand and use the rTMS technology, we did a scoping review to determine the rTMS-induced neural plasticity measured through fMRI in post-stroke patients with movement disorders.

## Method

### Scoping review

For this scoping review, we use the methodological framework developed by Arksey and O'Malley (26). Arksey and O'Malley suggest that there are five stages to a scoping review: (1) identifying the research question; (2) identifying relevant studies; (3) selecting studies; (4) charting the data; (5) collating, summarizing, and reporting the results.

### Identifying the research question

In this review, we will concentrate on the alteration of brain networks measured by fMRI after rTMS over M1 in stroke patients with movement disorders, and understand the brain mechanisms of rTMS. The scoping review may provide guidance for rTMS use as a therapeutic tool in movement disorders after stroke.

### Identifying relevant studies

PubMed, Embase, Web of Science, Embase, WanFang Chinese database, and ZhiWang Chinese database were accessed and searched from inception until December 2022. In addition, reference lists of relevant articles were screened to identify key articles that had been missed. The following search terms were entered using the Boolean operators AND/OR: “stroke”; “cerebrovascular accident”; “hemorrhagic stroke”; “ischemic stroke”; “cerebral stroke”; “brain vascular accident”; “transcranial magnetic stimulation”; “transcranial magnetic stimulations”; “repetitive transcranial magnetic stimulations”; “repetitive transcranial magnetic stimulation”; “transcranial magnetic stimulation, repetitive”; “rTMS”; “TMS”; “functional magnetic resonance imaging”; “fMRI”; “Functional MRI”; “fMRI”; “MRI”; “hemiplegia”; “motor deficit”; “dyskinesia”; “movement disorder”; and “motor dysfunction”; “motor deficits”; “movement disorders”; “motor dysfunction.” The search method was modified for each database following the example of PubMed (Table 1).

**Table 1:** Search strategy for PubMed.

**Search**	**Search term(s)**	**Results**
1	(Stroke OR Cerebral vascular accident OR Ischemic stroke OR hemorrhagic stroke OR Brain vascular accident OR Cerebral stroke)	469,217
2	(Transcranial Magnetic Stimulation OR Repetitive transcranial magnetic stimulation OR Repetitive transcranial magnetic stimulations OR Transcranial Magnetic Stimulations OR Transcranial Magnetic Stimulation, Repetitive OR rTMS OR TMS)	28,275
3	(Functional MRI OR fMRI OR Functional Magnetic Resonance Imaging OR MRI)	730,642
4	(Dyskinesia OR Movement disorder OR Motor dysfunction OR Hemiparesis OR Movement function OR Motor dysfunctions OR Movement disorders)	2,079,800
5	#1 AND #2 AND #3 AND #4	202

### Selecting studies

#### Inclusion and exclusion criteria

We formulated inclusion and exclusion criteria. The inclusion criteria were: (1) The study population included hemiplegia patients with ischemic or hemorrhagic stroke who performed functional assessment and underwent fMRI that was analyzed; (2) performed rTMS as the treatment; (3) fMRI as a tool to investigate brain activation before rTMS and after rTMS; (4) published in English or Chinese.

Exclusion criteria were: (1) Case studies, conference abstracts, systematic review, and meta-analysis; (2) After proper steps to locate the paper, the article was withdrawn or the complete text was not accessible.

#### Data management, screening, and extraction

**Figure 2:**
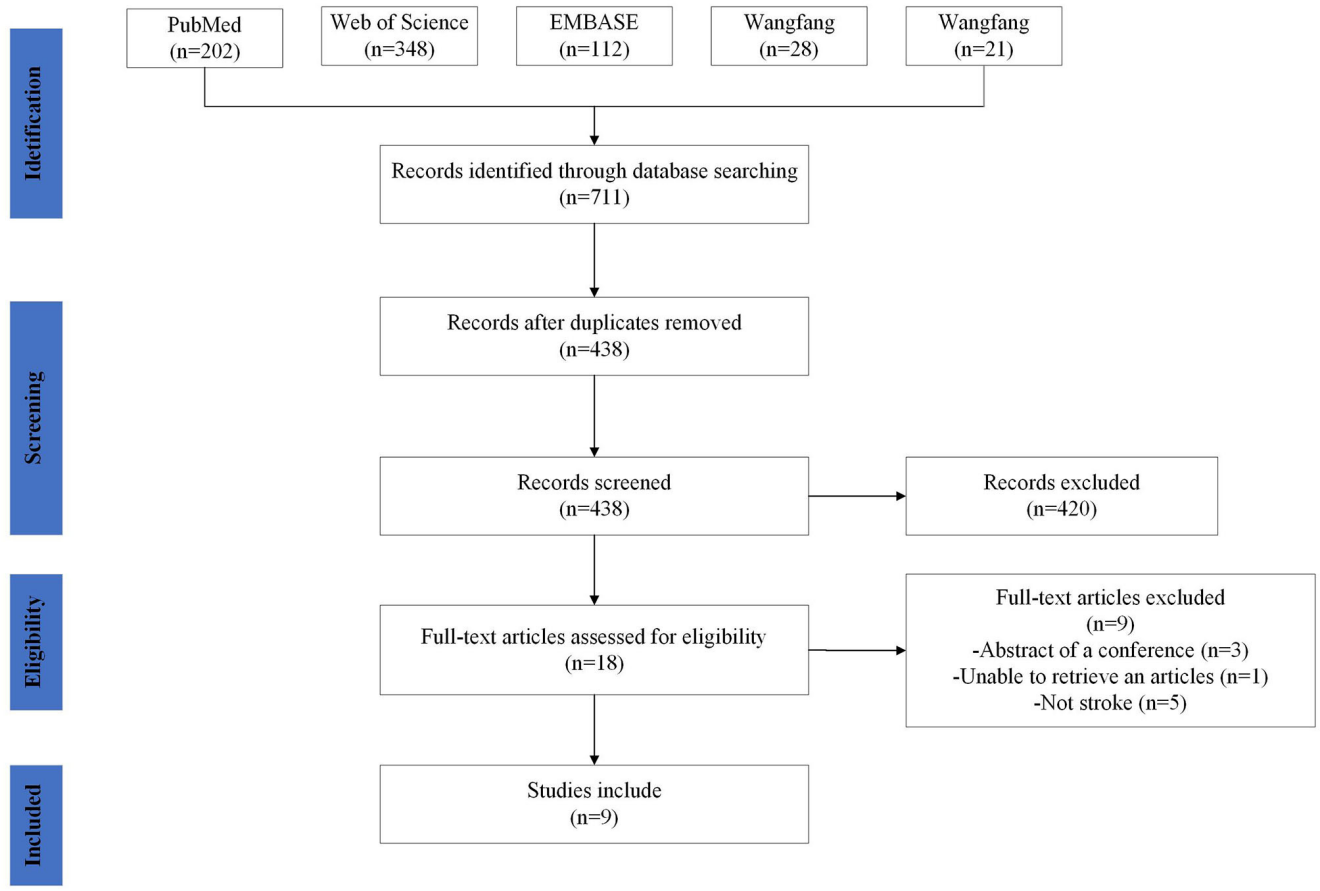
Study selection. Flow diagram showing the search strategy, the number of records identified, the excluded articles, and the studies eventually included. A total of nine studies were included.

The following phases were involved in study selection: titles and abstracts were reviewed for relevance by two reviewers according to the inclusion and exclusion criteria above. Then Full-texts were then screened. The two authors reached a consensus to determine whether this study should be included or excluded. If the two authors failed to reach consensus, a third author would have been consulted. Included articles were then examined to extract data. The process of identification, screening, eligibility, and inclusion of studies is pictured in Figure 2.

### Charting the data

#### Critical appraisal

Although a critical appraisal is not required by the scoping review, previous studies propose that the quality of evidence is an important part of this type of review (27). We choose the Downs and Black quality assessment checklist (28) to evaluate the level and quality of both randomized and nonrandomized controlled trials. According to the following cut-off points, we categorized the studies as excellent (26–28); good (20–25); fair (15–19); and poor ( ≤ 14) (29).

#### Data collection and synthesis

The data from each study were collected and categorized: each individual study (first author, year, and country of publication), study type, intervention delivered, outcome measurement and population characteristics. Regarding the rTMS protocol, the following stimulus parameters were extracted and recorded: targeted regions, stimulation intensity and frequency, number of TMS pulses per session, number of sessions, course and time. To evaluate whether and how rTMS modulated neural activity, we extracted the changes observed pre- and post-rTMS.

#### Collating, summarizing, and reporting the results

Seven hundred and eleven records were discovered in the database, 273 duplicate records were removed, and nine records passed the screening procedure' inclusion criteria. Figure 2 displays the screening procedure and the justifications for excluding research. The research contents are summarized in Table 2. In the included studies, the outcome measure was the Fugl–Meyer Assessment (FMA), Medical Research Council (MRC) scale, Wolf motor function test (WMFT), Motricity Index score (MI), Modified Rankin Scale (mRS) and Barthel Index (BI). The MRC scale has been the common and widely accepted assessment scale for muscle power, which grades muscle power on a scale of 0–5 (39). The WMFT consists of time and quality scales evaluating a set of 15 upper extremity functional tasks (40). As one of the most comprehensive quantitative evaluation indicators following stroke, FMA has been widely used in assessing reflex activity, motor control, and muscle strength, which consists of 33 items related to the motor function and the maximum exercise result is 66 points (41). The Motricity Index (MI) is an effective tool to assess stroke patients with motor dysfunction (42), which assessed muscle power by analyzing movements of all joints of extremities (43). mRS is a single item scale measuring the degree of disability or dependence in the daily activities for patients post-stroke (44). It is a well-designed scale, which is used to classify functionally independent levels with reference to pre-stroke activities. BI was is a frequently-used clinical assessment tool for daily living, thus reflecting motor function (45). As one of the most widely-used assessments of functional independence, BI is much more sensitive to changes in disability than the mRS (46).

**Table 2:** Characterization of studies that used rTMS in stroke.

**References**	**Country**	**Design: randomization/ blinding/sham**	**N (C, E) +**	**Mean Age (Years)**	**M/F**	**Disease duration**	**E/C intervention**	**rTMS protocol**	**Task-fMRI/Rs-fMRI**	**Other outcomes**
Ueda et al. (30)	Japan	NO/NO/NO	E: 1 Hz (*n* = 30)	59.7	19; 11	71.9 ± 47.2 (months)	Contra M1 LF-rTMS + OT	E: 1 Hz; 1,200 pulses; 90% rMT	Task-fMRI	FMA, WMFT
Juan et al. (31)	China	YES/YES/YES	C:Sham (*n* = 14) E1:LF rTMS (*n* = 17) E2:HF rTMS (*n* = 15)	C: 52 E1: 56 E2: 51	C: 12; 3 E1: 14; 3 E2: 12; 3	C: 6 ± 4 (days) E1: 4 ± 2 (days) E2: 4 ± 4 (days)	C: Sham rTMS + PT E1: Contra M1 LF-rTMS + PT E2: Ipsi M1 HF-rTMS + PT	C: the same parameters as E1, but with the coil rotated 90° away from the scalp; E1: 1 Hz; 1,200 pulses; 100% rMT; 5 consecutive days; E2: 10 Hz; 1,200 pulses; 100% rMT; 5 consecutive days	Rs-fMRI	FMA, MRC
Du et al. (32)	China	YES/YES/YES	C:Sham (*n* = 20) E1:LF rTMS (*n* = 20) E2:HF rTMS (*n* = 20)	C: 56 E1: 56 E2: 54	C: 16; 4 E1: 18; 2 E2: 14; 6	C: 4 ± 3 (days) E1: 4 ± 3 (days) E2: 5 ± 4 (days)	C: Sham rTMS+ PT E1: Contra M1 LF-rTMS + PT E2: Ipsi M1 HF-rTMS+ PT	C: the same parameters as E1, but with the coil rotated 90° away from the scalp; E1: 1 Hz; 1,200 pulses; 100% rMT; 5 consecutive days E2: 10 Hz; 1,200 pulses; 100% rMT; 5 consecutive days	Rs-fMRI	FMA, MRC
Guo et al. (33)	China	YES/YES/YES	C: Sham (*n* = 10) E1:LF rTMS (*n* = 12) E2:HF rTMS (*n* = 11)	C: 64.9 E1: 63.58 E2: 65.09	C: 5; 5 E1: 5; 7 E2: 5; 6	C: 5.1 ± 1.79 (days) E1: 5.42 ± 1.93 (days) E2: 6 ± 2.37 (days)	C: Sham rTMS+ PT E1: Contra M1 LF-rTMS + PT E2: Ipsi M1 HF-rTMS+ PT	C: the same parameters as E1 but without real stimulation E1: 1 Hz; 900 pulses; 90% rMT; 10 consecutive days E2: 10 Hz; 1,500 pulses; 90% rMT; 10 consecutive days	Rs-fMRI	FMA, BI
Chen et al. (34)	China	YES/YES/YES	C: Sham (*n* = 16) E1: HF + LF-rTMS (*n* =16) E2:HF rTMS (*n* =15) E3:LF rTMS (*n* = 16)	C: 59.813 E1: 53.250 E2: 56.800 E3: 59.688	C: 12; 4 E1: 10; 6 E2: 8; 7 E3: 11; 5	C: 7.938 (days) E1: 7.313 (days) E2: 6.667 (days) E3: 8.313 (days)	C: Sham rTMS+ PT E1: Ipsi M1 HF-rTMS+ Contra M1 LF-rTMS+ PT E2: Ipsi M1 HF-rTMS Contra M1 sham rTMS + PT E3: Contra M1 LF-rTMS + Ipsi M1 sham rTMS + PT	HF rTMS: 10 Hz; 600 pulses; 90% rMT; 5 days a week for 4 weeks LF-rTMS: 1 Hz; 600 pulses; 90% rMT; 5 days a week for 4 weeks; Sham rTMS: the same parameters as LF rTMS, but with the coil rotated 90° away from the scalp	Rs-fMRI	FMA, mRS
Wanni et al. (35)	Japan	NO/NO/NO	E:LF rTMS (*n* =70)	63	46; 24	43.5 ± 18.5 (months)	Contra M1 LF-rTMS + OT	E: 1 Hz; 1,200 pulses; 90% rMT; 12 days	Task-fMRI	FMA, WMFT
Grefkes et al. (36)	Germany	NO/NO/NO	E:LF rTMS (*n* = 11)	46	9; 2	1–3 (months)	Contra M1 LF-rTMS + OT	E: 1 Hz; 600 pulses; 100% rMT;	Task-fMRI	mRS, MRC, ARAT
Gottlieb et al. (37)	Germany	YES/YES/YES	C:Sham (*n* = 14) E: LF rTMS (*n* = 14)	C: 62 E1: 64	C: 5; 9 E: 3; 11	No mention	C:Sham rTMS+ OT+ PT E: Contra M1 LF- rTMS	E: 1 Hz; 1,200 pulses; 100% rMT; 5 days/week, 2 weeks C: the same parameters as E, the sham coil elicited the pulses in the opposite direction	Rs-fMRI	FMA, MAS
Tosun et al. (38)	Turkey	YES/YES/YES	C:Sham (*n* = 9) E1:LF rTMS (*n* = 9) E2:LF rTMS + NMES (*n* = 7)	C: 61.3 E1: 57.6 E2: 56	C: 5/4 E1: 6/3 E2: 3/4	C: 47.2 ± 41.1 (days) E1: 49.3 ± 43.6 (days) E2: 59.6 ± 58.3 (days)	C:PT E1: Contra M1 LF rTMS + PT E2: Contra M1 LF rTMS + NMES + PT	E: 1 Hz; 1,200 pulses; 90% rMT; 5 days/week, 4 weeks	Task-fMRI	FMA?BI

E, experimental group; C, control group; M, male; F, female; FMA, Fugl-Meyer Assessment scale; WMFT, Wolf Motor Function Test; MI, Motricity Index score; MRC, Medical Research Council; mRS, the modified Rankin scale score; BI, Barthel Index; rTMS, repetitive transcranial magnetic stimulation; fMRI, functional magnetic resonance imaging; M1, primary motor cortex; SMA, supplementary motor area; PMA, premotor area; NMES, neuromuscular electrical stimulation; PT, physical therapy; OT, occupational therapy.

## Result

### Search results

**Figure 3:**
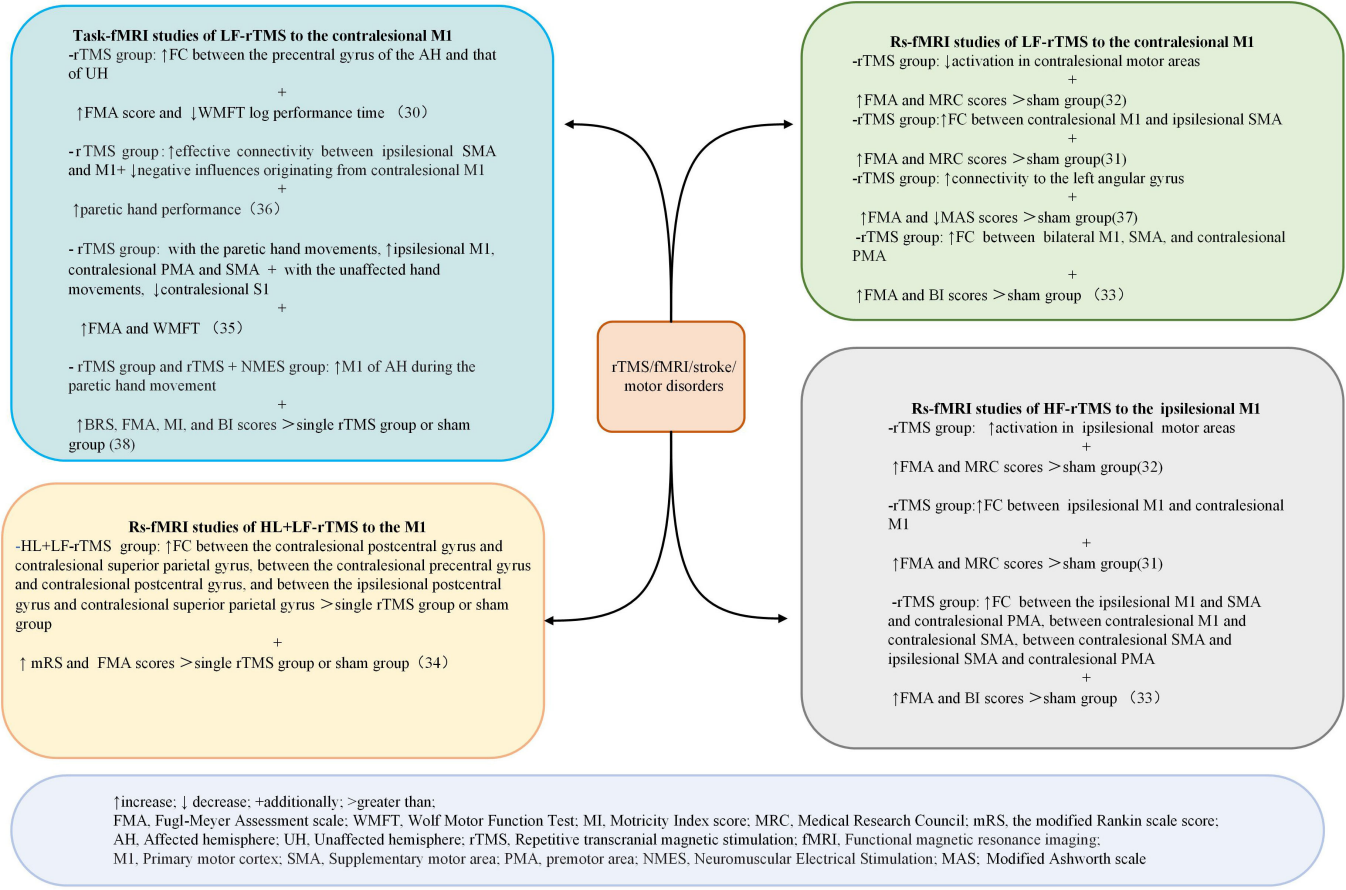
Summary of the main results of the included studies regarding the effects of rTMS on fMRI and motor outcomes in patients with stroke. ↑ increase; ↓ decrease; + additionally; >greater than. FMA, Fugl–Meyer Assessment scale; WMFT, Wolf Motor Function Test. MI, Motricitv Index score; MRC, Medical Research Council; mRS, the modified Rankin scale score; AH, affected hemisphere; UH, unaffected hemisphere; rTMS, repetitive transcranial magnetic stimulation: fMRI, functional magnetic resonance imaging; M1, primary motor cortex:; SMA, supp1ementarv motor area; PMA, premotor area; NMES. Neuromuscular Electrical Simulation; MAS, Modified Ashworth scale.

Databases searches identified 711 articles. After screening titles, abstracts, and full texts for eligibility, nine articles were included. Four studies were conducted in China, two in Japan, two in Germany, and one in Turkey. Five studies were RCTs, three were pre-post-test trials, and one was non-randomized controlled trials. A summary of the characteristics of the included papers is summarized in Table 2. Figure 3 shows the findings regarding the effects of rTMS on fMRI and motor performance in stroke survivors.

### Quality assessment

Table 3 displayed the outcomes of the quality evaluation of each study. Five studies were rated as having good quality and four as fair according to the Downs and Black criteria. The experimental hypothesis, the primary clinical features of the patients, the intervention techniques, and the key findings were all well reported in the papers under review. However, the studies met the requirements for the reporting section, but none provided the principal confounders in the groups and reported adverse events of intervention. Six studies failed to adhere to the requirement for blinded outcome assessment and participants blinded to treatment.

**Table 3:** Downs and Black checklist for quality assessment of included studies.

	**Ueda et al**.	**Du et al**.	**Juan et al**.	**Guo et al**.	**Chen et al**.	**Wanni et al**.	**Grefkes et al**.	**Gottlieb et al**.	**Tosun et al**.
**Reporting**									
Q1–Hypothesis/aim/objective clearly described	Yes−1	Yes−1	Yes−1	Yes−1	Yes−1	Yes−1	Yes−1	Yes−1	Yes−1
Q2–Main outcomes in Introduction or Methods	Yes−1	Yes−1	Yes−1	Yes−1	Yes−1	Yes−1	Yes−1	Yes−1	Yes−1
Q3–Patient characteristics clearly described	Yes−1	Yes−1	Yes−1	Yes−1	Yes−1	Yes−1	Yes−1	Yes−1	Yes−1
Q4–Interventions of interest clearly described	Yes−1	Yes−1	Yes−1	Yes−1	Yes−1	Yes−1	Yes−1	Yes−1	Yes−1
Q5–Principal confounders clearly described	UTD−0	UTD −0	UTD−0	UTD−0	No−0	No−0	No−0	No−0	No−0
Q6–Main findings clearly described	Yes−1	Yes−1	Yes−1	Yes−1	Yes−1	Yes−1	Yes−1	Yes−1	Yes−1
Q7–Estimates of random variability for main outcomes	Yes−1	Yes−1	Yes−1	Yes−1	Yes−1	Yes−1	Yes−1	Yes−1	Yes−1
Q8–All adverse events of intervention reported	No−0	No−0	No−0	No−0	No−0	No−0	No−0	No−0	No−0
Q9–Characteristics of patients lost to follow-up	Yes−1	Yes−1	Yes−1	Yes−1	Yes−1	Yes−1	Yes−1	Yes−1	Yes−1
Q10–Probability values reported for main outcomes	Yes−1	Yes−1	Yes−1	Yes−1	Yes−1	Yes−1	Yes−1	Yes−1	Yes−1
**External validity**									
Q11–Subjects asked to participate were representative of source population	UTD−0	UTD−0	UTD−0	UTD−0	UTD−0	UTD−0	UTD−0	UTD−0	UTD−0
Q12–Subjects prepared to participate were representative of source population	UTD−0	UTD−0	UTD−0	UTD−0	UTD−0	UTD−0	UTD−0	UTD−0	UTD−0
Q13–Staff/places/facilities study treatment was representative of source population	UTD−0	Yes−1	Yes−1	Yes−1	Yes−1	UTD−0	UTD−0	Yes−1	Yes−1
**Internal validity—bias and confounding**									
Q14–Study participants blinded to treatment	NO−0	Yes−1	Yes−1	NO−0	NO−0	NO−0	Yes−1	Yes−1	NO−0
Q15–Blinded outcome assessment	No−0	Yes−1	Yes−1	NO−0	UTD−0	NO−0	NO−0	Yes−1	Yes−1
Q16–Any data dredging clearly described	Yes−1	Yes−1	Yes−1	Yes−1	Yes−1	Yes−1	Yes−1	Yes−1	Yes−1
Q17–Analyses adjust for differing lengths of follow-up	Yes−1	Yes−1	Yes−1	Yes−1	Yes−1	Yes−1	Yes−1	Yes−1	Yes−1
Q18–Appropriate statistical tests performed	Yes−1	Yes−1	Yes−1	Yes−1	Yes−1	Yes−1	Yes−1	Yes−1	Yes−1
Q19–Compliance with interventions was reliable	Yes−1	Yes−1	Yes−1	Yes−1	Yes−1	Yes−1	Yes−1	Yes−1	Yes−1
Q20–Outcome measures were reliable and valid	Yes−1	Yes−1	Yes−1	Yes−1	Yes−1	Yes−1	Yes−1	Yes−1	Yes−1
Q21–All participants recruited from the same source population	Yes−1	Yes−1	Yes−1	Yes−1	Yes−1	Yes−1	Yes−1	Yes−1	Yes−1
Q22–All participants recruited over the same time period	UTD−0	UTD−0	UTD−0	UTD−0	Yes−1	UTD−0	UTD−0	Yes−1	Yes−1
Q23–Participants randomized to treatment(s)	No−0	Yes−1	Yes−1	No−0	Yes−1	No−0	No−0	Yes−1	Yes−1
Q24–Allocation of treatment concealed from investigators and participants	No−0	Yes−1	Yes−1	No−0	Yes−1	No−0	No−0	Yes−1	Yes−1
Q25–Adequate adjustment for confounding	No−0	No−0	No−0	No−0	No−0	No−0	No−0	No−0	No−0
Q26–Losses to follow-up taken into account	Yes−1	Yes−1	Yes−1	Yes−1	Yes−1	Yes−1	Yes−1	Yes−1	Yes−1
**Power**									
Q27–Power analysis to detect treatment effect at significance level of 0.05	Yes−1	Yes−1	Yes−1	Yes−1	Yes−1	Yes−1	Yes−1	Yes−1	Yes−1
TOTAL	16	21	21	17	20	16	16	22	21
Classification	Fiar	Good	Good	Fair	Good	Fair	Fair	Good	Good

Yes−1; No−0; Unable to determine UTD−0.

### Rs-fMRI studies of HF-rTMS to the ipsilesional M1

**Figure 4:**
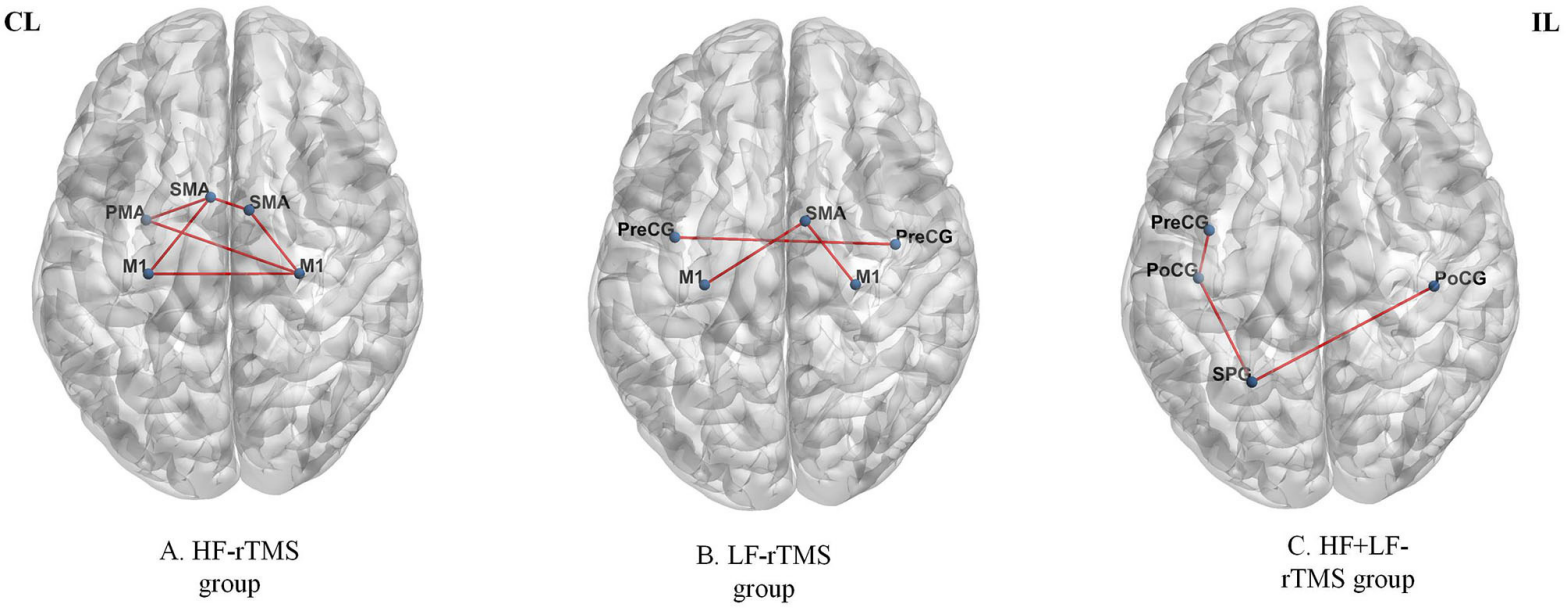
Significant functional connectivity changes after rTMS. The red edge represents an increase in the strength of connection. HF, high frequency; LF, low frequency; CL, contralesional side; IL, ipsilesional side; M1, primary motor cortex; SMA, supplementary motor area; PMA, premotor area; PreCG, precentral gyrus; PoCG, postcentral gyrus; SPG, superior parietal gyrus. The figure was drawn using BrainNet Viewer Software (http://nitrc.org/projects/bnv/).

**Figure 5:**
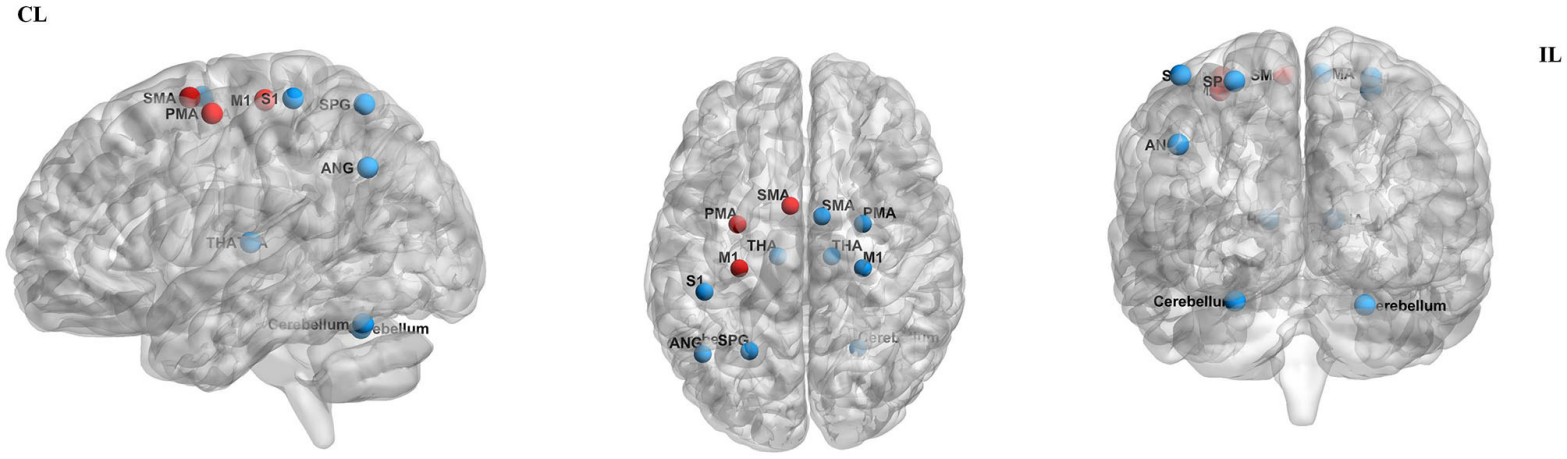
Significant activation of the brain areas after rTMS. Areas of significantly decreased activity are shown in red dots, while areas of significantly increased activity are shown in blue dots. CL, contralesional side; IL, ipsilesional side; M1, primary motor cortex; SMA, supplementary motor area; PMA, premotor area SPG, superior parietal gyrus; THA, thalamus; SI, primary somatosensory cortex; ANG, angular gyrus. The figure was drawn using BrainNet Viewer Software (http://nitrc.org/projects/bnv/).

By measuring the effects of the HF-rTMS (10 Hz) applied in the ipsilesional M1 on BOLD signals, Du et al. (32) found that HF-rTMS increased BOLD signal in the ipsilesional motor areas. Motor performance was observed in conjunction with fMRI changes. The effects of HF-rTMS (10 Hz) and LF-rTMS (1 Hz) were contrasted by Juan et al. (31). When applied the 10 Hz in the ipsilesional M1 they found an increase in resting-state functional connectivity (FC) between the bilateral M1, which has a positive relationship with motor performance. Guo et al. (33) assessed the effect of functional reorganization following rTMS in stroke survivors as well as the differences between HF-rTMS and LF-rTMS. In the HF-rTMS group, they found higher FC between ipsilesional M1 and contralesional premotor area (PMA), which suggests that HF-rTMS can increase the FC of the ipsilesional motor network and enhance the motor functions. Significant functional connectivity changes after rTMS are summarized in Figure 4. Significant activations of the brain areas after rTMS are summarized in Figure 5.

### Rs-fMRI studies of LF-rTMS to the contralesional M1

Du et al. (32) show LF-rTMS reduced brain excitability and fMRI activation in contralesional motor region. These changes were accompanied by improved motor activity. Juan et al. (31) reported 1 Hz rTMS applied to contralesional M1 resulted in enhanced FC between contralesional M1 and ipsilesional SMA. Motor improvement evaluated using the FMA and MRC scale was significantly enhanced in the real rTMS group compared with the sham group. Gottlieb et al. (37) found that connectivity to the left angular gyrus increased after LF-rTMS over M1. The modified Ashworth scale (MAS)score was reduced and the FMA score improved in the LF-rTMS group, suggesting motor improvement. Significant functional connectivity changes after rTMS are summarized in Figure 4. Significant activations of the brain areas after rTMS are summarized in Figure 5.

### Rs-fMRI studies of HL + LF-rTMS to the M1

Chen et al. (34) found that inhibitory-facilitatory rTMS treatment induced greater increases in FC between multiple brain regions in comparison to the other groups using single-course or sham rTMS, resulting in great improvements in motor function. Significant functional connectivity changes after rTMS are summarized in Figure 4. Significant activations of the brain areas after rTMS are summarized in Figure 5.

### Task-fMRI studies of LF-rTMS to the contralesional M1

Ueda et al. (30) found significant FC changes in bilateral cerebral hemispheres after LF-rTMS during task-fMRI. According to Grefkes et al. (36), 1-Hz rTMS has suppressive effects on ipsilesional M1 and facilitates more efficient motor processing in the contralesional hemisphere, as shown by the improved coupling of SMA and M1. Wanni et al. (35) show significant activations were seen in the ipsilesional PMA, M1, and thalamic-cortical regions with the paretic hand movements after rTMS. However, significant activations in the contralesional primary somatosensory cortex (S1), superior parietal cortex, and bilateral cerebellum with unaffected hand movements after the intervention were observed. There was a considerable improvement in FMA and WMFT from pre- to post-rTMS. Tosun et al. (38) reported that greater activation of the affected M1 was observed during the movements of the paretic hand in most patients of the TMS group and TMS + NMES group. Significant functional connectivity changes after rTMS are summarized in Figure 4. Significant activations of the brain areas after rTMS are summarized in Figure 5.

## Discussion

### Neural plasticity

**Figure 6:**
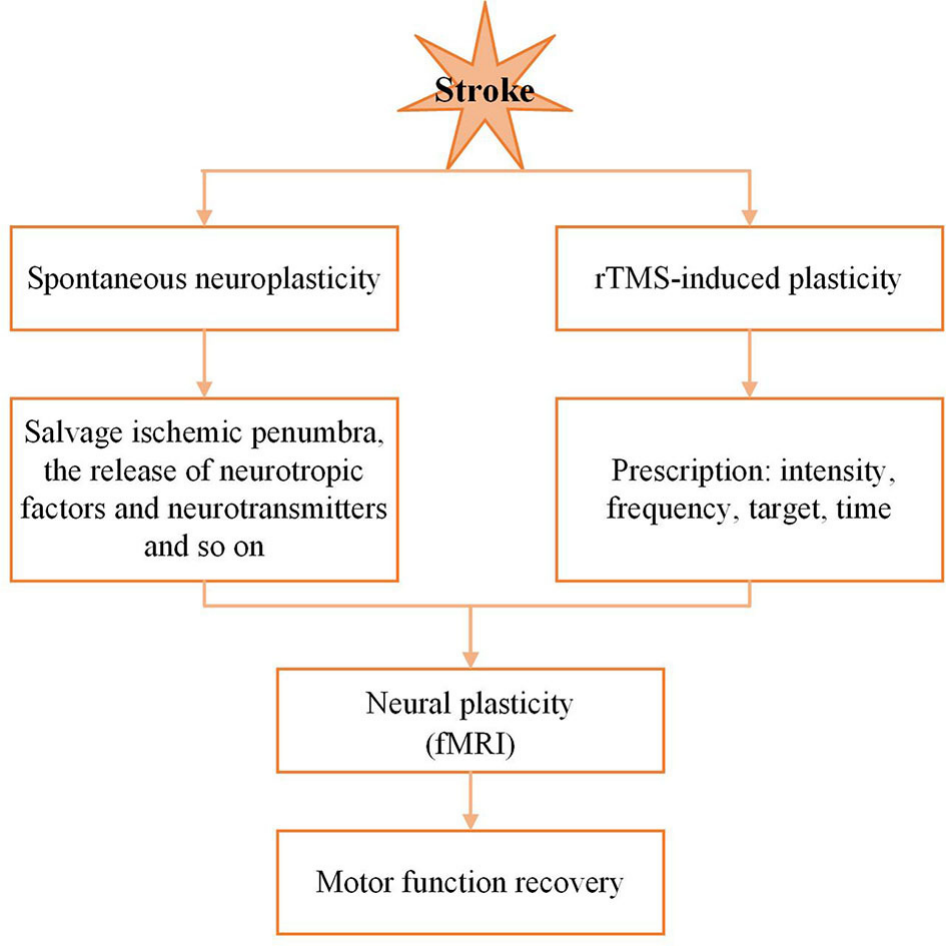
Neuroplastic mechanisms of motor recover after stroke.

There is growing evidence to suggest an association of rTMS with the induction of neural plasticity to promote stroke recovery (47). “Neural plasticity” refers to the ability of modification of the nervous system in response to suffering and to adapt following trauma by remodeling its structure, functions, or connections (48). The processes of neural plasticity include altered excitability of neuronal circuits, reorganization by using redundant connections, and formation of new functional connections, which may partly compensate for the lost function. Based on neuroplasticity research, motor function recovery after stroke is related with spontaneous neuroplasticity changes and rTMS-induced plasticity (49). During the post-stroke recovery stage, the spontaneous remodeling neural networks were accompanied by functional recovery (50, 51). It is possible that these spontaneous changes in neuroplasticity are associated with pathophysiological mechanisms, such as salvage ischemic penumbra by revascularization, the release of neurotropic factors and neurotransmitters (52). However, these spontaneous neuroplasticity changes have a limited effect on stroke recovery (53). After stroke, rehabilitation can promote dynamic processes due to increase effective neuronal information input, promote neural repair and functional compensation (54). rTMS was identified to be a successful rehabilitation method for improving stroke recovery by promoting neuroplasticity. The application of fMRI provides a good understanding of the mechanisms involved. Recently, plasticity-induced rTMS was combined with fMRI to map plastic aftereffects (55). The neuroplastic mechanisms of motor function recovery after stroke can see the Figure 6.

### Plasticity changes during recovery

Neural plasticity takes place at a very early stage after stroke, lasts for some time, and involves brain regions remote from the lesioned site. Schulz et al. (56) proved that the corticospinal tract fibers not only originating from the M1 and the ipsilesional PMA and SMA contributed to motor function after stroke. Pathologically, damage to the brain related to motor function, such as M1, PMA, SMA, and S1 (57), contribute to hemiplegia following stroke. Activation in M1 of the affected hemisphere is reduced and the activation is relocated toward the PMA and SMA during movements of the affected hand after stroke (58, 59). By comparing a shift in sensorimotor cortical excitability in both hemispheres, it was found the activation of the contralesional sensorimotor cortex increases accompanying movements of the paretic hand in most post-stroke (60). After a stroke, there are alterations in neural activity in bilateral cerebral hemispheres, which can be beneficial, but can also contribute for maladaptive recovery (61). Conventionally, LF-rTMS or HF-rTMS is effective in improving motor functions by rebalancing hemispheres' the excitability (32).

There have been many studies on local and global functional connections analysis. The most consistent conclusion is that the interhemispheric connection between regions involved in motor function has changed significantly, and the degree of connectivity is related to motor functions. In the early post-stroke period, motor network resting-state connections progressively decrease (62). fMRI studies demonstrated that the patients with reduced the functional connections between bilateral M1 are more severe in motor performance and with increased functional connection between the ipsilesional M1 and the contralesional thalamus, SMA and middle frontal gyrus at the stage of acute stroke was beneficial to the motor recovery (63). The decreased interhemispheric functional connections between homologous motor areas were associated with the degree of motor function in the acute phase (64), While the enhanced functional connections had a positive correlation with the spontaneous recovery of motor function in the weeks to months after stroke (62). The patients with good motor function recovery showed functional connections between homologous motor regions came back to normal levels in the stable phase after stroke. However, the patients with poor motor function recovery found that functional connections remained low (65, 66). Therefore, it is necessary to seek alternative approaches that strengthen the natural plasticity of the sensorimotor system, which is particularly effective in enhancing motor improvement. In rTMS studies, functional connectivity (FC) evaluation is of value because it enables the evaluation of rTMS effects.

### The bimodal balance–recovery model

The interhemispheric competition, assuming the presence of mutual, balanced inhibition between the hemispheres in the healthy people, is found to be altered after stroke (12). Damage to one hemisphere in stroke results not only from neuronal loss within the affected hemisphere but also from the downregulation of remaining neurons within the affected hemisphere resulting in increased inhibition of the affected hemisphere by the unaffected hemisphere (12). That is to say that both the stroke itself and the excessively high interhemispheric inhibition from the contralesional hemisphere result in a double impairment of the ipsilesional cortex. Based on TMS therapy (30), ipsilesional motor areas play an active and vital role in stroke recovery. Therefore, the downregulation of contralesional cortical excitability may be helpful for enhancing motor recovery. Researchers and clinicians have used rTMS to restore the interhemispheric balance to gain motor improvement by either enhancing the activtion of the lesioned hemisphere (38) or inhibiting the healthy hemisphere.

However, it has been found that HF-rTMS given over the M1 of the unaffected side is more effective than LF-rTMS when patients with significant harm to the affected side (67). The significance of the contralesional hemisphere in motor recovery following stroke has been thoroughly investigated in the past few years. According to Ueda et al. (30), the cortical activity of the ipsilesional and the contralesional motor areas changes synchronously during movements of the paralyzed hand after rTMS in stroke survivors with moderate disability. fMRI research found the contralesional PMA was significantly activated during the movement of the paralyzed hand in stroke survivors (68). A meta-analysis (69) shows that consistently activated regions involved the contralesional M1, the bilateral PMA, and SMA in stroke patients compared to healthy controls. The extent of this activation is likely to be influenced by the size and location of the injured region, being greatest in stroke survivors with the greatest impairment. From what has been discussed above, the enhanced activation of the PMA, SMA, and the contralesional hemisphere make up for the lost function in stroke survivors with severe motor impairment. Thus, the vicariation model (12) was presented, which assumes activity in residual networks helps stroke people recover function lost by damaged areas. Similarly, rTMS can support residual motor function following stroke by inducing positive compensatory effects of contralesional mirror motor regions (67).

The vicariation and interhemispheric competition are the two models of functional recovery, which hold opposite views and represent different neuromodulatory treatments. According to interhemispheric competition, stroke recovery would be facilitated by the downregulation of the contralesional hemisphere since it would free the injured hemisphere from aberrant inhibition by the ipsilesional hemisphere. The vicariation model, however, contends that such a tactic would impede compensating activity in the contralesional hemisphere. However, neither the interhemispheric competition model nor the vicariation model could account for all stroke recovery. Therefore, a novel theory called the bimodal balance-recovery model (12) was put out to account for the various contributions made by the contralesional hemisphere to post-stroke motor recovery. The biphasic recovery model of the transcallosal suppression model and the compensatory model may be the neurophysiological basis of functional recovery after stroke. Due to the high level of structural preservation, the interhemispheric competition model is more helpful for stroke survivors with moderate motor impairment. In contrast, patients with little structural reserve may find the vicariation model more helpful in predicting a recovery. Excessive activation in contralesional M1 and nonprimary motor areas can be seen in the early phase after stroke, suggesting recruiting of these brain regions after the cerebral vascular accident (70). This is in accordance with the result that shows overactivity in the ipsilesional and contralesional PMC, SMA, parietal cortex, and contralesional M1 during movements of the hemiplegic hand (71). In a word, bilateral cerebral hemispheres may both participate in the restoration of motor functions brought about by rTMS intervention. It is necessary to conduct more researches to examine whether rTMS therapy can benefit more stroke patients using individualized stimulation techniques.

### RTMS for modulating plasticity following stroke

#### RTMS improves neural activity

Pathologically, specific brain damage associated with motor functions, such as M1, PMA, and SMA, can lead to motor disorders. The simultaneous activation of bilateral sensorimotor cortices would strengthen coherence of cortical activity, which is a crucial neurophysiological mechanism promoting interaction via transcallosal connections between the related brain regions. rTMS has been considered an effective strategy promoting the recovery of motor functions, which may cause alterations in brain activity and connectivity in local and distant areas after stroke (8). To balance out both sides of cortical excitability, HF-rTMS and LF-rTMS have been used, promoting or hindering the affected and unaffected M1, respectively (32). In both cases, rTMS can correct the maladaptive brain plasticity induced by stroke or enhance adaptive brain plasticity. Based on the fMRI findings, significant clusters in the bilateral cerebellum were observed during the unaffected hand movements after rTMS, suggesting the cerebellum play a role in stroke recovery (35). The cerebellum maintains many neural connections with the motor cortex, which controls motor skills such as motor coordination, fine motor, and motor learning (72). Moreover, the fMRI findings also found activations in bilateral thalamocortical circuits are associated with affected hand motions after rTMS (35). The thalamus served as a relay station for the sensory-motor route, sending relevant sensory and motor information to the cortex (73). Activations of the corpus callosum after rTMS in the included study (35) are in general consistent with previous research that indicated similar change in the affected hand movements after intervention (74). Another study demonstrated that changes in the structure of the corpus callosum are related to transcallosal inhibition and upper limb dysfunction in chronic stroke (75). The regaining of motor function in stroke patients may be influenced by the corpus callosum, which functions as a connector for information between brain hemispheres. Activation of motor cortices triggers brain plasticity, which may lead to enhanced cortico-subcortical connections and cortico-subcortical pathways, which are associated with functional recovery after stroke (53, 76). In these included studies, we found that rTMS reorganizes not only motor-related networks, M1, SMA, and PMA but also the cerebellum, thalamus as well as the corpus callosum.

#### RTMS improves functional connectivity

Functional connectivity can quantify the functional integration of different brain areas by correlating brain activity in order to detect neural interactions between regions, which are quite convincing (77). Not only are many brain activities related to particular brain regions, but also to connections between different brain regions. Damage to the brain's structural components may result from the localized neurological condition, and this damage may have an impact on how distant brain areas function (78). Stroke may result in neurological impairment by affecting localized, specific areas of the brain, but more evidence proves that the network effects resulting from the loss of connections between distant brain areas are important reasons for neurological impairment (5). Restoration of interhemispheric functional connectivity was only seen in stroke survivors who had recovered well, not in those who had recovered poorly (79), suggesting that interhemispheric functional connectivity in stroke patients is a significant indicator established (80). Therefore, increasing motor network connections after stroke may be particularly useful for promoting motor recovery.

Furthermore, not only does rTMS affect the stimulated functional network, but also physically or functionally related remote brain areas. A large body of studies suggests that increased connections between major motor areas in bilateral cerebral hemispheres may underlie rTMS-mediated functional gains. By altering connections between motor areas in both the stimulated and non-stimulated hemispheres, rTMS may be used to improve motor function by reversing pathological alterations in the functional network architecture. The majority of interhemispheric connections, including M1, S1, and PMA, were involved in motor performance. Interhemispheric functional connections between ipsilesional M1 and contralesional M1 were significantly decreased after stroke (81). The studies (31, 33) indicated that increased functional connections of ipsilesional/contralesional M1 could be served as the main target of motor rehabilitative regimes for stroke patients. Guo et al. (33) demonstrated the increased functional connections between ipsilesional M1 and contralesional PMA were associated with motor recovery after rTMS, suggesting contralesional PMA may be contributed to mediating motor recovery after stroke. Grefkes et al. (36) proved LF-rTMS enhanced coupling of SMA and M1, constituting a significant mechanism for better motor function. Some rodents studies confirmed improving function is mostly dependent on the restoration of neural networks in the ipsilesional hemisphere (82). Motor recovery is the result of a repair of ipsilesional effective connectivity between SMA and M1 as well as a reduction of abnormal transcallosal impacts. The activation of SMA is linked to the attention to intention, which is essential for voluntary motor movement, and it is an important compensatory area of movement (83). Gottlieb et al. (37) found the connectivity between the motor cortex and the angular gyrus increased after the LF-rTMS, and the angular gyrus is responsible for controlling upper limb motions (84). A previous study has shown that the postcentral gyrus is the primary somatosensory cortical center (85). The preservation of connectivity between the ipsilesional M1 and the contralesional postcentral gyrus indicates better motor performance (86). Therefore, the activation of contralesional postcentral gyrus is a significant part in the reorganization of motor function. Upper limb movement is closely related to M1 region located in the precentral gyrus (87). A better prognosis of motor function is associated with activation of precentral gyrus and postcentral gyrus in stroke patients (88). In an earlier study, the superior parietal gyrus was shown to be part of space object positioning and visual-motor coordination (89). Chen et al. (34) confirm increased functional connectivity in the contralesional precentral gyrus, postcentral gyrus, and the parietal gyrus in the recovery of motor function. Changes in interhemispheric connectivities are associated with an imbalance between the contralesional hemispheres and ipsilesional hemispheres after stroke onset. Interhemispheric connectivities are increased by the rebalancing of bilateral hemispheric networks during the recovery period. These functional connectivity changes were linked to the recovery of motor function restoration and could be targeted for neurorehabilitation interventions following stroke. Although LF-rTMS and HF-rTMS have opposite effects on cortical activity of the directly stimulated region, these two frequencies of rTMS tend to increase functional connectivity. Especially, most studies using LF-rTMS protocol, reported increases in functional connectivity rather than reductions. Interhemispheric functional connection decreased sharply, and increased significantly in parallel with motor recovery after rTMS, suggesting the importance of interhemispheric functional connection in the recovery of motor function after stroke.

In summary, these studies have demonstrated that rTMS can promote interhemispheric connectivity by increasing activation in ipsilesional regions, enhancing excitatory connectivity from the ipsilesional to the contralesional brain regions, and reducing stroke-induced transcallosal inhibition to facilitate facilitates the recovery of motor performance.

### RTMS protocols

It has been shown that the long-lasting after-effects of plasticity-inducing rTMS can easily impact human behavior. The combination of rTMS with fMRI provides a unique opportunity to elucidate the mechanistic basis for such behavioral effects. Thus, this combination allows us to gain insight into the local and distant effects of different interventions and provides a means of addressing changes in functional connectivity that underlie potential behavioral effects. The currently accepted strategy to promote the recovery of motor function after stroke is to either apply HF-rTMS (31–33) to the M1 region of the ipsilesional hemisphere or apply LF-rTMS (30–33, 35–38) to the M1 region of the contralesional hemisphere. In clinical practice, the combination of HF-rTMS and LF-rTMS is relatively rare, but some studies have shown that the combination of the two is feasible and safe, and the therapeutic effect is better than the single application (90). In comparison to sham stimulation or single-course rTMS, coupled inhibitory-facilitatory rTMS significantly improved motor function.

rTMS is a noninvasive neuromodulation technique whose variability in the stimulation intensity, frequency, duration, and target location influence modulatory effects (91). The HF-rTMS stimulation and the LF-rTMS stimulation both can improve motor function by rebalancing motor cortex excitability and regulating connectivity in patients after stroke (32). Du et al. (25) found that rTMS could dramatically enhance motor function, and this enhancement was strongly connected with changes in the motor cortex excitability, and LF-rTMS may have more profound effects than HF-rTMS. The HF-rTMS group offers better advantages for the functional connection recombination of the motor network on the ipsilesional brain, bringing greater benefits for the recovery of motor disorders (33). A meta-analysis revealed that HF-rTMS is marginally more efficient than LF-rTMS (92). Another meta-analysis, however, revealed a different outcome (93). Chen et al. (34) also found that the combined application of low-frequency and high-frequency rTMS had a synergistic effect on improving motor function and cortical excitability of patients. At present, the optimal effective frequency of rTMS is still uncertain. The 2014 European Guidelines for the treatment of rTMS indicate that both LF-rTMS or HF-rTMS can be used to restore motor function after stroke (94). The application of LF-rTMS shows level A evidence for motor stroke in the post-acute phase, as well as level C evidence in the chronic phase. While the application of HF-rTMS shows level A evidence in the post-acute phase (15).

### Limitations and future direction

The summary of current evidence suggests that rTMS may change neural plasticity, which was associated with movement improvement after stroke. This scoping review revealed some important findings. First, in the process of searching the literature, we discovered most rTMS studies only focused on the functional connectivity changes in motor network, and few studies have involved deep brain areas. However, many movement diseases, including Parkinson's disease and stroke, are associated with the damage of the thalamus, putamen, cerebellum, and other subcortical regions (95, 96). As the included studies in the review seldom focused on whole-brain connectivity, but rather specific regions of interest, more researches should pay attention to the whole-brain connectivity. In the future, the regulation mechanism of rTMS on deep brain regions can be explored by analyzing the changes in functional connectivity in cortical and subcortical regions, which will provide a reference for the treatment of these diseases. Second, it should be highlighted that the number of studies applied fMRI to investigate the aftereffects of rTMS on the functional brain network following stroke is relatively limited, and the effects of rTMS, in particular, have seldom been mapped with task-based fMRI. Future research should concentrate on mapping the effects of rTMS on task-related activity and connectivity (97). Third, the last three decades have seen the progress of neuroimaging technology, which allow people to examine neuroplasticity noninvasively. However, each of them has its merits and demerits. Multimodal neuroimaging technology can merge information and overcome some of the limitations of the stand-alone neuroimaging method. Multimodal fusion technology permits more granular inspection of the underlying mechanisms of plastic after-effects of rTMS protocols. However, studies using multimodal fusion technology to explore neural mechanisms underlying rTMS intervention are relatively rare. Therefore, incorporating multimodal neuroimaging technology into clinical trials will help expand our knowledge of neural mechanisms underlying rTMS intervention and ultimately tailor the therapeutic use of rTMS. Fourth, fMRI studies have confirmed the role of bilateral M1 areas after a stroke, indicating functional connectivity between the areas and functional connectivity between M1 and other brain areas is associated with motor recovery (63). Although M1, as an important brain area responsible for planning and performing motor functions, is the ideal stimulated brain area, stimulating rather the PMA has many benefits (98). When brain damage is severe, PMA of the contralesional hemispheres exerts an excitatory effect on the M1 affected hemisphere. When brain lesions are small, PMA of the contralesional hemispheres exerts an inhibitory effect (99). SMA is related to complicated motions and the programming of these complicated motions, which is a crucial issue to address in motor skill recovery. In addition, for patients with motor dysfunction accompanied by cognitive impairment or depression, the left dorsolateral frontal lobe can be selected as the stimulation target (100), and the recovery of motor function can be promoted by improving the cognitive and depression symptom. To date, studies have focused mostly on the motor cortex, whereas other parts of the brain are still underrepresented. Future researches may deepen the topic to concomitantly evaluate different targets areas. Lastly, none of the studies perform fMRI during rTMS, and a fMRI scan was usually performed at baseline and after rTMS that lasted several weeks. This approach is limited in its sensitivity to capture post-stimulation effects, and is likely to fail to map the immediate consequences of the stimulation. For solving this problem, simultaneous rTMS-fMRI, in which rTMS is applied during fMRI scan, permits research into the immediate effects of rTMS and the underlying processes of rTMS-mediated neuronal regulation.

## Conclusion

The studies reviewed above strongly suggest that rTMS can be used to modulate disturbed cortical networks thereby improving motor function. As a noninvasive imaging method, fMRI is commonly used in brain activation studies. It works based on the changes in blood flow caused by neuronal activity. In response to neuronal activity, blood flow to the region can be accelerated to meet the increased demand for oxygen (101). fMRI studies can show a changing process of recovery of motor function. fMRI studies can start in the first few days poststroke and continue for several months to a year poststroke (102). The integration of fMRI and rTMS represents a powerful tool for manipulating and observing neural activity, which can unravel the mechanisms of TMS-mediated neural modulation. We deeply think that creating innovative techniques for the effective treatment of post-stroke movement disorders requires a thorough knowledge of the neural mechanisms underlying recovery from movement disorders.

## Data availability statement

The original contributions presented in the study are included in the article/supplementary material, further inquiries can be directed to the corresponding authors.

## Author contributions

SC and RX: conception and design of the work, acquisition, analysis, interpretation of data for the work, and drafting the manuscript. PW, PL, and WF: supervising the manuscript and final approval of the manuscript to be submitted. YZ and PL: revising the manuscript. All authors contributed to the article and approved the submitted version.
